# Fairness and accountability of AI in disaster risk management: Opportunities and challenges

**DOI:** 10.1016/j.patter.2021.100363

**Published:** 2021-11-12

**Authors:** Caroline M. Gevaert, Mary Carman, Benjamin Rosman, Yola Georgiadou, Robert Soden

**Affiliations:** 1 Department of Earth Observation Science, Faculty ITC, University of Twente, Enschede, Overijssel 7514AE, the Netherlands; 2 Department of Philosophy, Faculty of Humanities, University of the Witwatersrand, Johannesburg, Gauteng 2000, South Africa; 3 School of Computer Science and Applied Mathematics, Faculty of Science, University of the Witwatersrand, Johannesburg, Gauteng 2000, South Africa; 4 Department of Urban and Regional Planning and Geo-Information Management, Faculty ITC, University of Twente, Enschede, Overijssel 7514AE, the Netherlands; 5 Department of Computer Science and School of the Environment, University of Toronto, Toronto, ON M5T 1P5, Canada

**Keywords:** disaster risk management, DRM, geospatial, artificial intelligence, AI, values, accountability

## Abstract

Disaster risk management (DRM) seeks to help societies prepare for, mitigate, or recover from the adverse impacts of disasters and climate change. Core to DRM are disaster risk models that rely heavily on geospatial data about the natural and built environments. Developers are increasingly turning to artificial intelligence (AI) to improve the quality of these models. Yet, there is still little understanding of how the extent of hidden geospatial biases affects disaster risk models and how accountability relationships are affected by these emerging actors and methods. In many cases, there is also a disconnect between the algorithm designers and the communities where the research is conducted or algorithms are implemented. This perspective highlights emerging concerns about the use of AI in DRM. We discuss potential concerns and illustrate what must be considered from a data science, ethical, and social perspective to ensure the responsible usage of AI in this field.

## The bigger picture

Artificial Intelligence (AI) is increasingly being used in disaster risk management applications to predict the effect of upcoming disasters, plan for mitigation strategies, and determine who needs how much aid after a disaster strikes. The media is filled with unintended ethical concerns of AI algorithms, such as image recognition algorithms not recognizing persons of color or racist algorithmic predictions of whether offenders will recidivate. We know such unintended ethical consequences must play a role in DRM as well, yet there is surprisingly little research on exactly what the unintended consequences are and what we can do to mitigate them. The aim of this perspective is to call researchers working on fairness, accountability, and transparency to work with DRM and local experts—so we can ensure that disaster mitigation and relief is accountable, considers local values, and is not unintentionally biased.

## Teaser

Disaster risk management increasingly relies on artificial intelligence algorithms to help societies mitigate and overcome the adverse impacts of disasters. Yet, there is little research on the ethical implications of using these algorithms. This perspective illustrates concerns voiced by DRM practitioners and highlights the need for research on how to adapt technical solutions for mitigating bias and define new accountability relations. Finally, we emphasize the need to include local experts in prioritizing ethical values.

## Introduction

Climate change and population growth in urban areas are increasing the risk of persons and infrastructure to disasters. In 2021 alone, disasters affected more than 98.4 million people, including more than 15,000 deaths and an estimated economic loss of USD 171.3 billion.^1^ Disaster risk management (DRM) aims to help societies recover from disasters and prepare for and mitigate the impact of future disasters. Geospatial data play a key role in DRM by mapping populations and infrastructure exposed to disasters, identifying areas at risk, and planning emergency responses.^2^ Remotely sensed imagery from satellites or drones can extract proxies to analyze pre-disaster vulnerability and resilience and post-disaster damage and recovery.^3^ As with other domains, DRM has recognized the potential of artificial intelligence (AI) algorithms to rapidly and accurately process data, and is now using AI to develop more accurate risk models and prioritize the distribution of disaster aid.^4^

Despite the great potential of AI to support DRM processes, practitioners express concerns regarding the ethical and responsible usage of these algorithms. How can a practitioner be certain that their risk model is not biased against the most vulnerable societal groups in a city? How are accountability relationships in disaster aid influenced by the introduction of AI when algorithm developers are far from the disaster and unfamiliar with the local context? A plethora of guidelines on ethical or responsible usage of AI is emerging, each promoting slightly different values and definitions. See, for example, the extensive comparison of leading guidelines for “principled” AI by Fjeld et al.^5^ The ethical or responsible usage of AI often circles around a handful of important concepts, including fairness, accountability, and transparency (FAccT). *Fairness or non-discrimination* generally refer to the absence of bias in datasets and algorithms. Research shows that algorithms trained on biased datasets fail to recognize historically excluded groups in society (e.g., Barocas and Selbst^6^). *Accountability* relates to the mechanism or process through which a forum can hold an actor to justify their actions (see Olson et al.,^7^ pp. 60–61). In an AI context accountability then turns to discussions on who is the actor (the developer of the algorithm? the organization deploying the algorithm?), and how their actions can be justified. The latter requires *transparency or explicability*, and much research is focused on exactly how we can shed light on the inner workings of complex AI algorithms to enable this justification. Such concerns regarding FAccT are being thoroughly investigated by social and data scientists for applications of AI in domains such as criminal justice and education,^8^ but not yet in the field of DRM and geospatial sciences.

The objective of this perspective is to call researchers interested in FAccT and other ethical considerations of AI to look toward the field of DRM. The perspective first provides a brief introduction to FAccT research and then delineates potential concerns observed by DRM experts to emphasize the demand for FAccT research and solutions in the DRM community. We then continue to describe the technical aspects of geospatial data, which is prominent in DRM applications, and how its peculiarities require tinkering with the technical tools to audit data and preserve FAccT principles in DRM workflows. Thirdly, we emphasize how DRM applications highlight the need for inclusivity, including vulnerable populations in holding distant, big data algorithms, and international corporations to account, and inclusivity in the way that FAccT research is conducted and values are defined. Finally, we provide recommendations on how to start integrating these two fields more closely.

## Fairness, accountability, and transparency

The rise of AI has galvanized the integration of automated algorithms into decision-making systems. However, there is also an increasing concern regarding the ethical implications of these systems. Research into the ethical aspects of AI systems is known as ethical AI, FAT, FAT/ML, FAT∗, or FAccT. FAT refers to the three concepts: FAccT. The addition of ML refers to applications related to machine learning, and ∗ is a type of wildcard emphasizing that other ethical components, such as inclusivity, power, and justice are also considered in this field of research. FAccT is the latest denomination adopted by one of the most influential conferences in the field: the ACM Conference on Fairness, Accountability, and Transparency (ACM FAccT).^9^

Research in the field of FAccT is slightly paradoxical as it aims to develop technical solutions to audit and ensure ethical values in AI workflows, although the values themselves are ambiguous by nature. For example, *fairness* generally refers to a lack of bias in an AI system against a certain individual or group, but there are multiple definitions of fairness utilized in the FAccT literature. Some adopt a statistical approach based on similar performance in classification metrics, e.g., that different cultural groups should have the same chances of achieving a defined algorithmic output. Other approaches test whether a predefined sensitive variable (e.g., gender) influences the output of an algorithm.^10^ To complicate matters even further, although many definitions of fairness achieve similar results, they are sensitive to data variability and some definitions may achieve conflicting outputs.^11^ Indeed, Kleinberg et al.^12^ go so far as to prove theoretically that some commonly accepted definitions of fairness are incompatible. It is not the purpose of this perspective to give an extensive comparison of different fairness metrics or other ethical values researched in the FAccT field. For this, the curious reader is encouraged to consult excellent reviews on fairness (e.g., Verma and Rubin^10^), accountability (e.g., Wieringa^13^), and transparency (e.g., Mittelstadt et al.^14^) in the literature. However, for the context of this perspective it is important to recognize why research in the field of FAccT requires such close collaboration between data science and humanities, as well as knowledge of the context in which the algorithm will operate.

The FAccT community is made up of researchers from machine learning, statistics, data science, law, and social sciences, as well as interested industry bodies, such as Google, IBM, and Microsoft, as illustrated by the participants in the annual ACM FAccT conference.^9^ Nevertheless, a significant critique of FAccT research in general is that, even if perfect technical solutions for FAccT values could be embedded into AI systems, there is generally a lack of understanding regarding the social, cultural, and political environment in which these systems are deployed.^15^^,^^16^ Indeed, FAccT conferences remain dominated by American institutes and mainly white male authors,^17^ although there are strong efforts to increase diversity. These concerns highlight the timeliness of our call for FAccT researchers to consider investigations in the DRM domain. This manuscript emphasizes rich opportunities provided by DRM to develop the theoretical framework of FAccT, new technical solutions, and boost inclusivity.

## Potential concerns articulated by the DRM community

Several broad categories of concern arise when introducing AI into DRM. Drawing on recent work in the DRM community,^18^ we can highlight three of them here.

Firstly, *bias* is a recurring topic of discussion among responsible AI practitioners. Given the global scope of DRM activities, and wide variations in quality and coverage of geospatial data, bias is indeed a significant risk. For example, call detail records generated by mobile phones may be used to estimate population sizes before and after a disaster,^19^ but may underestimate vulnerable populations who have no access to cell phones.^20^ Often conceived of as statistical error driven by choices around sampling data by data scientists and technical experts, bias in the responsible AI literature is often viewed more broadly.^21^ Thus, bias can also be the result of algorithm design or decisions around metrics used to evaluate a particular phenomenon. While some technical approaches to addressing bias exist, completely eliminating bias in algorithms is impossible, and, as some have argued,^22^^,^^23^ exclusive focus on reducing bias in AI systems may distract from other, more important, interventions.

A second concern relates to *transparency and accountability*. The introduction of AI techniques and their associated complexity into disaster risk modeling processes may reduce the ability of government, the public, and other important stakeholders to meaningfully participate in DRM. Even modelers themselves have reported that, in some circumstances, their ability to understand and evaluate the outputs of AI models has decreased in comparison with traditional approaches to disaster risk modeling.^18^^,^^24^ Indeed, the field of explainable AI was created largely to address this issue. For example, Behl et al.^25^ used explainable AI to investigate the potential limitations of an algorithm trained to process Twitter data to identify people’s needs after a disaster. However, in general the “explanations” provided by explainable AI are dissonant from how human beings typically construct explanations.^26^ With reduced transparency of complex AI models comes increased challenges in ensuring that experts and decision-makers involved in DRM are accountable to the communities that they serve.^27^

Finally, the *hype* and *inflated expectations* that surround AI at the moment may lead to one or more interrelated problems. Most directly, untested or immature AI tools may be used in safety-critical situations for which they are not yet ready. This may in turn draw needed resources and attention away from more suitable approaches or encourage over-reliance on tools that are not fit for purpose. Disasters are often viewed as opportunities for innovation, but in the hands of uncareful or unscrupulous developers this can be a recipe for harm.^28^ A good example of avoiding hype is given by forecast-based financing, introduced by the Red Cross Red Crescent movement to release disaster response funding before the impact of a disaster. The release of funds is contingent on the predicted impact of a pending disaster, which can be modeled by AI. However, a validation committee not only checks the validity of the proposed AI model, but also whether simpler, expert-based systems would be more appropriate.^27^

## Adaptations of technical solutions to geospatial data

Taking bias as an example, we can show how DRM applications can benefit from the technical solutions developed by FAccT researchers and how DRM applications provide opportunities for FAccT researchers to develop new solutions. DRM applications often depend on geospatial data. Satellite and drone imagery provide snapshots of the world below and can be used to generate maps of buildings and important infrastructure. Mobility and social media data can provide insights into the movement of citizens. Hazard models, such as flood models, show the spatial extent of the area at risk. Unfortunately, both the DRM and FAccT communities pay little attention to biases that are embedded in geospatial data.

There is a well-known lack of up-to-date (geospatial) data in low- to middle-income countries (LMICs) compared with high-income countries (HICs) (e.g., the Center for Humanitarian Data^29^). OpenStreetMap (OSM) in conjunction with humanitarian mapping efforts have aimed to improve the disparity in data availability of HICs versus LMICs, and yet a significant gap remains as humanitarian mapping efforts seem to focus on areas of past disasters, areas containing local mapping communities, and areas of interest for specific stakeholders, such as development agencies.^30^ Little information is available for rural or unprioritized areas. Similarly, mobility and social media data excludes those without a digital footprint, such as disadvantaged communities with limited access to digital technology.

Many AI algorithms are used to process this kind of geospatial data. Back in the 1950s, methods consisted of spatial interpolation through kriging (more recently, Gaussian processes) or simple decision trees. Expert-based machine-learning systems became popular in the 1980s and this shifted to data-driven machine-learning techniques such as support vector machines in the 1990s and random forests at the turn of the millennium. The last ten years have been heavily influenced by developments in computer vision, and deep learning techniques are now being widely applied to geospatial data.^31^ These techniques and algorithms are leading to unprecedented classification accuracies and show much promise for DRM applications. However, they also suffer the same vulnerabilities identified in other domains using these algorithms, such as susceptibility to bias.

FAccT researchers are developing technical tools to identify and mitigate bias in such AI algorithms. Some of these solutions can directly be applied to DRM applications. For example, Suresh and Guttag^32^ illustrate the role of historical bias, representation bias, measurement bias, aggregation bias, evaluation bias, and deployment bias in machine-learning algorithms. These same biases can easily be identified in DRM workflows. Historical data used to train hazard models may not take the impacts of climate change into account^33^ (i.e., historical bias) and conflicting definitions used to identify vulnerable populations may grossly underestimate the population living in poverty^34^ (i.e., measurement bias).

In other cases, adaptations are needed when applying developed techniques to geospatial data and DRM applications. Auditing for biases often depends on the identification of sensitive attributes. Well-known examples of representation bias and evaluation bias include the underrepresentation of races or gender in training and evaluation data. However, it is not clear which types of groups may be underrepresented in geospatial data and thus how these data should be audited to check for potential biases.

Sometimes such sensitive attributes can be directly identified in DRM applications. For example, household surveys or other demographic data that contain sensitive data may be utilized. The use of social media for disaster warnings and post-disaster damage assessment may contain biases on gender, income level, and minority groups.^35^

In other cases, the geospatial data used for DRM does not directly specify sensitive attributes but can indirectly capture information that is related to socio-economic or cultural groups. For example, the body of research on informal settlement mapping often relates the physical characteristics of buildings to the socio-economic status of its inhabitants. Characteristics identifiable in remotely sensed imagery, such as small buildings, low-quality roofing materials, irregular street patterns, and narrow streets are strongly related to impoverished living conditions.^36^ Maps produced through machine learning could therefore be audited regarding which types of objects (such as building types) may be underrepresented in addition to the geographical distribution of the training data. Close communication with stakeholders and the DRM application would define which physical characteristics or objects should be considered as possibly sensitive.

Another important factor relates to the distance of the remote mapper from the local context and the power bias in deciding what is to be mapped. Research by LMICs is grossly underrepresented in the DRM research community.^37^ Similarly, the perception of which type of information is important for addressing DRM issues is often defined in HICs and may overlook important local norms and contexts,^38^ which are crucial for solving complex social issues. Visual analyses of drone imagery in slum areas by local residents illustrates that the perceptions of sensitive objects varied greatly in different areas^39^ and emphasizes that interpreting remote sensing imagery is context dependent. The lack of understanding of local context may exclude local assets and values from being represented in geospatial data utilized for DRM and thus inhibits the ability of these data to support the development of locally effective mitigation measures.

The example of bias thus illustrates both how DRM applications can benefit from the technical solutions developed by FAccT researchers and how DRM applications provide opportunities for FAccT researchers to develop new solutions. We turn now to considering how questions of accountability and inclusivity tie in with DRM, first through the lens of humanitarianism and then by taking a step back to consider the very way in which values are defined. Again, we aim to highlight the interplay between FAccT considerations and DRM.

## Accountability in neoclassical humanitarianism: Epistemic justice

DRM as a field of research and practice is the progeny of classical humanitarianism, which stands for the life-saving relief assistance and protection historically provided by the International Committee of the Red Cross in conflict situations. DRM became practically synonymous with new humanitarianism in the early 2000s with the distinction between the two fields becoming increasingly blurred in the digital and algorithmic era, especially after 2010. The merging of the two fields was accompanied by calls for accountability, itself a value that has had different meanings over time. Tracing the historical evolution of humanitarian action, especially after it merged with DRM, as well as the related meanings of accountability may help us identify and salvage valuable concepts from classical humanitarianism—a symbol of global moral progress and a humanizer of the world^40^—in the era of machine learning.

**Figure 1:**
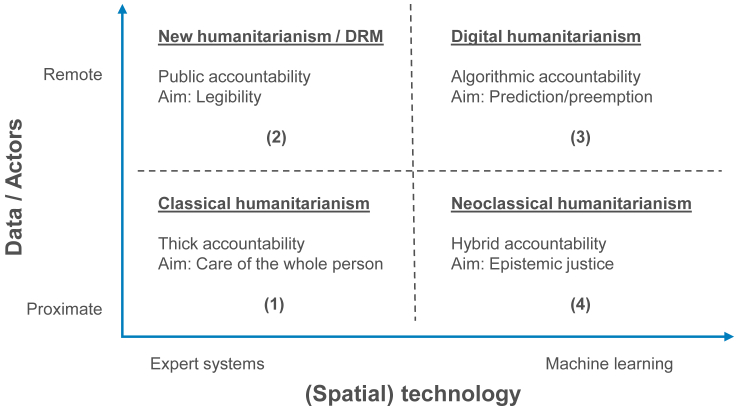
An overview of the evolution of humanitarian action in four stages

Figure 1 captures four stages in the evolution of humanitarian action. The horizontal axis ranges from expert systems replicating human decision rules to machine learning that generates predictive models using techniques such as probabilistic reasoning. We use the term “expert” broadly to refer to an expert not as a special kind of person but to every person as a special kind of expert, especially with respect to their own problems.^41^ The vertical axis ranges from actors in face-to-face proximity with beneficiaries to actors very remote from beneficiaries—for example, corporate philanthropists, commercial geospatial and mobile phone companies, self-organizing voluntary networks of digital humanitarians, universities, and international space agencies. The same vertical axis ranges from data collected in face-to-face interactions in the field to data collected by remote digital humanitarians, satellites, drones, mobile phone companies, and the like. The two axes span a space that allows us to conceptualize the evolution of humanitarian action over time.

In classical humanitarianism (cell 1) the aim is the “care of the whole human person, all of her or him.”^42^ The thickly accountable humanitarian has empathy and compassion for victims of crises, makes herself vulnerable and reachable in the field, must earn the respect of victims and understand others’ suffering. New humanitarianism (cell 2) foregrounds public accountability—the right of empowered citizens to hold state actors to account for failures to make the territory and the people legible and governance rules enforceable, for when disaster strikes. DRM scholars define public accountability as:

A relationship between an actor and a forum, in which (a) the actor has an obligation to explain and justify his or her plans of action and/or conduct, (b) the forum may pose questions, require more information, solicit other views, and pass judgement, and (c) the actor may see positive or negative formal and/or informal consequences as a result (Olson et al.,^7^ p. 61).

The 2010 Haiti earthquake is a case in point. As Olson et al.^7^ argue, the Haitian state allowed Port au Prince to evolve in a way that created extreme vulnerabilities. Port au Prince was not legible to the state; there was no record of land-use planning. There was no record of an enforced building code. Development “actors” in Haiti felt no obligation:

to pass any of their siting or building plans by any ‘forum’ (which in effect did not exist anyway), nor were there any negative formal or informal consequences for their not doing so. This complete lack of public accountability was a recipe for the massive urban human vulnerability that only required a major (not even a great) earthquake to become a national catastrophe. (p. 64).

In digital humanitarianism (cell 3) machine learning generates predictive models and early action^27^ and accountability becomes algorithmic.^13^ For instance, cash transfers to flood victims may result in faster, more secure aid before the flood event but also give access to vast amounts of data to actors with non-humanitarian intentions. Disasters complexify algorithmic accountability, especially when human rights or data protection legislation is absent or weakly enforced. Largely untested and non-consented humanitarian interventions are deployed with inferior standards to analyze the need and evaluate the effectiveness of interventions, while the power asymmetry between humanitarian actors and beneficiaries is radically increased.

This brings us to what we may tentatively call neoclassical humanitarianism (cell 4) and the open question “How to reinterpret proximity in the age of machine learning?” or “Whether we can salvage the principle of ‘caring for the whole person, all of him or her’ with machine learning and, if yes, how?” Machine learning may be used to make important decisions about humanitarian subjects without their being able to observe, understand, participate in, or respond to information gathered or assumptions made about them. One possible answer is to reduce, even eliminate, epistemic injustice.^43^ Epistemic injustice has both an ethical and an epistemic significance—someone is wronged in their capacity as a knower, as an epistemic subject. Imagine a flood victim or a refugee accused of concocting stories to improve their chances for aid or asylum. Imagine a flood victim or a refugee unable to process let alone communicate her traumatic experiences coherently to other people. Getting swept up in the hype of the potential uses of AI could mean that algorithmic predictions may be given a higher weight than the subject’s own version of events, assuming that the subject was asked in the first place. Aiming for epistemic justice in DRM research and practice could lead to a new understanding of (a hybrid?) accountability in the machine-learning era.

## Diverse values: Ensuring inclusivity at all levels

At the end of the previous section, we asked whether we can salvage the principle of humanity, of “caring for the whole person, all of him or her,” with machine learning and, if yes, how. Another possible answer is to promote inclusivity not just at the level of reducing epistemic injustice by recognizing others as knowers, but at the level of incorporating diverse systems of values by recognizing others as valuers. At this level, we are not necessarily looking specifically at issues of bias, accountability, and fairness arising within the operations of AI and machine learning but rather at the ethical development and implementation of such technologies, where inclusivity arises in how objectives are framed and priorities are balanced (see Carman and Rosman^44^). This is an area where DRM throws certain values, such as autonomy and privacy, into stark relief for researchers of FAccT principles to engage with, as we discuss in this penultimate section before moving on to some recommendations.

As already discussed, there is (1) an underrepresentation of research by LMICs in the DRM research community, (2) a need to incorporate local norms and context, and (3) the perception of which type of information is important is often defined by HICs. Similar points can be made at the level of ethical frameworks for ethical AI more generally.

With the first, there is again an underrepresentation of ethical research by LMICs in the development of international frameworks and guidelines for ethical AI, even though ethical research in other applied fields, such as bioethics, is thriving. For instance, even though discussions for ethical AI within African contexts typically draw on international frameworks (see, for instance, Microsoft^45^), there are very few African voices contributing to the development of these frameworks in the first place.

With the second, overlooking the local norms and contexts even at the level of what ethical values we prioritize can have an impact. The former director of Médecins Sans Frontiers, Roy Braumann, for instance, narrates a story of how medical workers involved in an emergency food supply in famine-stricken Uganda prioritized giving food to women and children as the most vulnerable. However, it transpired that food was in fact being redistributed to the elderly, in line with local customs and values that promote respecting social order (see Hellsten,^46^ p. 73). By overlooking local customs and values, the program risked being ineffective, but in terms of “caring for the whole person,” it also raises challenges about imposing outside values on others. Within a context of many LMICs where DRM interventions are focused, countries frequently with turbulent histories of colonialism, the further imposition of outside values risks being a form of neo-colonialism. As Cletus Andoh writes with regard to another area of practical implementation where values may be imposed uncritically, bioethics within Africa, “assimilating Western values and ideologies into Africa can give rise to a situation of self-dehumanisation and outright self-subversion both in terms of dignity and self-esteem” (Andoh,^47^ p. 69).

With the third, we can draw attention to how the type of information deemed important is often defined by HICs. Take, for instance, a recurring principle that is prioritized in frameworks for ethical AI and which is often drawn on to frame objectives is a principle of respecting autonomy: the principle that “individuals have a right to make decisions for themselves” (Floridi et al.,^48^ p. 697). Such a principle is critical in the field of geospatial data, where questions regarding privacy and what counts as personal information over which one has decision-making power are central. Yet, this is a principle that has been widely challenged in other areas of research as not being representative of more communitarian values, such as those held by many cultures across Africa.^49^^,^^50^ In such contexts, community-oriented decision-making processes may in fact be promoted and valued, rather than individualistic ones. This is a case of a principle and its associated values being deemed important from an imported context, where in fact critical reflection and application is required.

For instance, even on a highly personal topic such as reproductive health, research has shown that many women in developing countries, largely in communalistic contexts, prefer to involve their partners in making decisions regarding involvement in the research,^51^ which is at odds (but accommodated as an exception) with international health research ethics. In a similar fashion, communication about important topics, such as climate change, may need to adopt participatory methods,^52^ a consideration that can be extended to communications about drone usage. Recognizing that such a principle may not have universal relevance, “caring for the whole person” would require not treating diversions from an individualistic conception and valuing of autonomy as a rule with exceptions, at risk of ostracizing alternative cultural values.

As the use of geospatial data throws issues of privacy into tangible relief, the emerging space of the use of this data and AI systems within DRM creates an exciting field where diverse ethical values can be tackled head on, hopefully providing input and guidance for the FAccT community at large. This is yet another area where we need to be cautious of being swept up in the hype generated by the potentials of AI, at risk of causing harder to measure harms, such as those arising from epistemic injustice or failing to care for the “whole person.”

## Conclusions and outlook

In this perspective, we emphasize the opportunities of turning researchers interested in FAccT and other ethical considerations of AI toward DRM and geospatial data. There is a clear call from DRM specialists for expertise on how to deal with bias, transparency, accountability, hype, and inflated expectations.

Bias in geospatial settings can be partially addressed through the technical workflows that are being developed to audit algorithms for bias. However, it is not clear which potential biases to audit for and the consideration of bias in geospatial applications has been, until now, mainly limited to considering the geographic distribution of data. Further research is required to carefully consider how the way geospatial data are obtained and deployed, because DRM applications may induce biases. Furthermore, identifying biases requires a detailed understanding of the local context to identify which sensitive groups the algorithm should be audited for and which values the algorithm should prioritize. Including experts of the local context, both community members affected by natural disasters or academic experts capable of elucidating local values and understandings for algorithm designers, is needed as a starting point to reduce epistemic injustice, promote humanitarian values, and to increase accountability of machine-learning algorithms toward local actors. Governments could play a significant role in facilitating these interactions and leveraging community voices in international dialogues. These interactions should focus on concretizing key algorithmic decision moments where social values are embedded in algorithms, identifying sensitive groups that the algorithm should be audited for bias, and how to clearly communicate the results and uncertainties of DRM algorithms to reduce inflated expectations.

At a fundamental level, we also need to examine conceptually how differing sets of values can inform how we define problems. Such considerations regarding diverse ethical worldviews and values are not new and we can draw on existing literature from other fields as a starting point. Having a better understanding of context can inform how best to proceed with framing problems and objectives. For example, Gevaert et al.^39^ investigated how the understanding of the value of something like privacy and what counts as personal data may change depending on background values. Okoliko and de Wits^52^ demonstrated how communicating about climate change in African contexts effectively, recognizing communitarian values, may require participatory methods, a good example of how respecting and incorporating values on the ground may impact communication.

The field of DRM can draw on discussions and research on issues such as FAccT within AI at large but, as we have hoped to show, it also can cast a new light on familiar but also new aspects of ensuring ethical and inclusive AI.

## References

[1] CRED & UNDRR (2021).

[2] Kemper H., Kemper G. (2020). Sensor fusion, GIS and AI technologies for disaster management. Int. Arch. Photogramm. Remote Sens. Spat. Inf. Sci. ISPRS Arch..

[3] Ghaffarian S., Kerle N., Filatova T. (2018). Remote sensing-based proxies for urban disaster risk management and resilience: a review. Remote Sens.

[4] GFDRR (2018).

[5] Fjeld J., Achten N., Hilligoss H., Nagy A., Srikumar M. (2020). Principled artificial intelligence: mapping consensus in ethical and rights-based approaches to principles for AI. SSRN Electron. J..

[6] Barocas S., Selbst A.D. (2016). Big data’s disparate impact. Calif. L. Rev..

[7] Olson R.S., Sarmiento J.P., Hoberman G. (2011). Establishing public accountability, speaking truth to power and inducing political will for disaster risk reduction: ‘Ocho Rios + 25. Environ. Hazards..

[8] O’Neil C. (2016).

[9] A.C.M. FAccT (2021). https://facctconference.org.

[10] Verma S., Rubin J. (2018). Proceedings of the International Workshop on Software Fairness.

[11] Friedler S.A., Scheidegger C., Venkatasubramanian S., Choudhary S., Hamilton E.P., Roth D. (2019). Proceedings of the Conference on Fairness, Accountability, and Transparency.

[12] Kleinberg J., Mullainathan S., Raghavan M. (2016). Inherent trade-offs in the fair determination of risk scores. ArXiv.

[13] Wieringa M. (2020). Proceedings of the 2020 Conference on Fairness, Accountability, and Transparency.

[14] Mittelstadt B., Russell C., Wachter S. (2019). Proceedings of the Conference on Fairness, Accountability, and Transparency.

[15] Selbst A.D., Boyd D., Friedler S.A., Venkatasubramanian S., Vertesi J. (2019). Proceedings of the Conference on Fairness, Accountability, and Transparency.

[16] Article 19 (2018).

[17] Acuna D.E., Liang L. (2021). Proceedings of the 2021 AAAI/ACM Conference on AI, Ethics, and Society.

[18] Soden R., Wagenaar D., Tijssen A. (2021).

[19] Yu M., Yang C., Li Y. (2018). Big data in natural disaster management: a review. Geosciences.

[20] Pestre G., Letouzé E., Zagheni E. (2020). The ABCDE of big data: assessing biases in call-detail records for development estimates. World Bank Econ. Rev..

[21] Barocas S., Crawford K., Shapiro A., Wallach H. (2017). 9th Annual Conference of the Special Interest Group for Computing, Information and Society.

[22] Noble S.U. (2018).

[23] West S.M. (2020). Redistribution and rekognition. Catal. Fem. Theory, Technoscience..

[24] Wagenaar D., Curran A., Balbi M., Bhardwaj A., Soden R., Hartato E., Sarica G.M., Ruangpan L., Molinario G., Lallemant D. (2020). Invited perspectives: how machine learning will change flood risk and impact assessment. Nat. Hazards Earth Syst. Sci..

[25] Behl S., Rao A., Aggarwal S., Chadha S., Pannu H.S. (2021). Twitter for disaster relief through sentiment analysis for COVID-19 and natural hazard crises. Int. J. Disaster Risk Reduct..

[26] Miller T. (2019). Explanation in artificial intelligence: insights from the social sciences. Artif. Intell..

[27] Van den Homberg M.J.C., Gevaert C.M., Georgiadou Y. (2020). The changing face of accountability in humanitarianism: using artificial intelligence for anticipatory action. Polit. Gov..

[28] Sandvik K.B., Jacobsen K.L., McDonald S.M. (2017). Do no harm: a taxonomy of the challenges of humanitarian experimentation. Int. Rev. Red Cross..

[29] The Centre for Humanitarian Data (2020). https://reliefweb.int/sites/reliefweb.int/files/resources/StateofData2020.pdf.

[30] Herfort B., Lautenbach S., Porto de Albuquerque J., Anderson J., Zipf A. (2021). The evolution of humanitarian mapping within the OpenStreetMap community. Sci. Rep..

[31] Dramsch J.S. (2020). 70 years of machine learning in Geoscience in review. ArXiv.

[32] Suresh H., Guttag J.V. (2020). A framework for understanding unintended consequences of machine learning. ArXiv.

[33] Lavell A., Oppenheimer M., Diop C., Hess J., Lempert R., Li J., Field C.B., Barros V., Stocker T.F., Qin D., Dokken D.J., Ebi K.L. (2012). Managing the Risks of Extreme Events and Disasters to Advance Climate Change Adaptation. A Special Report of Working Groups I and II of the Intergovernmental Panel on Climate Change (IPCC).

[34] Lucci P., Bhatkal T., Khan A. (2018). Are we underestimating urban poverty?. World Dev..

[35] Zhang C., Yang Y., Mostafavi A. (2021). Revealing Unfairness in social media contributors’ attention to vulnerable urban areas during disasters. Int. J. Disaster Risk Reduct..

[36] Abascal Á., Rothwell N., Shonowo A., Thomson D.R., Elias P., Elsey H., Yeboah G., Kuffer M. (2021). “Domains of deprivation framework” for mapping slums, informal settlements, and other deprived areas in LMICs to improve urban planning and policy: a scoping review. Preprints.

[37] Orimoloye I.R., Ekundayo T.C., Ololade O.O., Belle J.A. (2021). Systematic mapping of disaster risk management research and the role of innovative technology. Environ. Sci. Pollut. Res..

[38] Abebe R., Aruleba K., Birhane A., Kingsley S., Obaido G., Remy S.L., Sadagopan S. (2021). Proceedings of the 2021 ACM Conference on Fairness, Accountability, and Transparency.

[39] Gevaert C.M., Sliuzas R., Persello C., Vosselman G. (2018). Evaluating the societal impact of using drones to support urban upgrading projects. ISPRS Int. J. Geoinf..

[40] Barnett M.N. (2013). Humanitarian governance. Annu. Rev. Poli. Sci..

[41] Hisschemöller M., Hoppe R. (1995). Coping with intractable controversies: the case for problem structuring in policy design and analysis. Knowl. Policy.

[42] Slim H. (2015).

[43] Fricker M. (2013). Epistemic justice as a condition of political freedom?. Synthese.

[44] Carman M., Rosman B. (2020). Applying a principle of explicability to AI research in Africa: should we do it?. Ethics Inf. Technol..

[45] Microsoft (2019).

[46] Hellsten S.K. (2008). Global bioethics: utopia or reality?. Dev. World Bioeth..

[47] Andoh C.T., Andoh C.T. (2011). Bioethics and the challenges to its growth in Africa. Open J. Philos..

[48] Floridi L., Cowls J., Beltrametti M., Chatila R., Chazerand P., Dignum V., Luetge C., Madelin R., Pagallo U., Rossi F. (2018). AI4People—an ethical framework for a good AI society: opportunities, risks, principles, and recommendations. Minds Mach..

[49] Chukwuneke F., Umeora O., Maduabuchi J., Egbunike N. (2014). Global bioethics and culture in a pluralistic world: how does culture influence bioethics in Africa?. Ann. Med. Health Sci. Res..

[50] Barugahare J. (2018). African bioethics: methodological doubts and insights. BMC Med. Ethics.

[51] Moodley K. (2007). Microbicide research in developing countries: have we given the ethical concerns due consideration?. BMC Med. Ethics.

[52] Okoliko D.A., de Wit M.P. (2020). From “communicating” to “engagement”: afro-relationality as a conceptual framework for climate change communication in Africa. J. Med. Ethic.

